# Why people matter in 21st century hydrology: lessons learnt from flood forecasting and warning

**DOI:** 10.1098/rsta.2024.0293

**Published:** 2025-07-31

**Authors:** Linda Speight

**Affiliations:** ^1^ School of Geography and the Environment, University of Oxford, Oxford, UK

**Keywords:** climate adaptation, decision making, flood warning, hydrology, people, risk communication

## Abstract

In a world facing the challenge of adapting to a rapidly changing climate, hydrologists have an important role in ensuring the risks from floods, droughts and polluted water are addressed. Despite ongoing scientific advances, and a growing focus on people-centred hydrology that supports solutions to global hydrological challenges, there remains a disconnect between hydrological expertise, policy and practice. The best hydrological models in the world are no use to society if they do not result in effective action. The devastating impacts from recent unprecedented flood events in Europe highlight the urgency of addressing this challenge. Reflecting on the communication of flood risk during extreme events offers valuable insights into why people matter to closing the gap between hydrological expertise and society. People are considered here in respect to four themes: people and hydrological science, people and hydrology education, people and hydrological decision making and people within the discipline of hydrology. As a discipline hydrology must better articulate why it matters to people to allow it to overcome the challenges of complacency and complexity in risk communication. Hydrologists themselves must be flexible and innovative, developing new skills sets to support continued interdisciplinary collaborations and expertise to better integrate people into hydrology.

This article is part of the Royal Society Science+ meeting issue ‘Hydrology in the 21st century: challenges in science, to policy and practice’.

## 1. Introduction

Our climate is changing rapidly, hydrological events are becoming more extreme and occurring more frequently. There is increasing evidence that observed changes in the hydrological cycle are affecting people and ecosystems, especially the most vulnerable communities [1]. The ability to manage hydrological risk in the long term, and to forecast, warn and take preventative action in advance of events is an essential component of any climate adaption strategy. The World Weather Attribution reports for the year 2024 have documented the hydrological impacts of Hurricanes Helena and Milton hitting Florida (USA) in quick succession, the interaction of Typhoon Gaemi with the monsoon in the Philippines, exceptionally low rainfall and high temperatures, leading to ongoing droughts in South America and Europe and 10 extreme rainfall events covering all continents [2]. Events that have had devastating impacts on people and communities and are unprecedented in terms of their scale, intensity, characteristics or impacts [3]. All were made more likely due to climate change and all highlight the increasing exposure to hydrological risk and a growing adaption gap caused, in part, by financial constraints, poor governance and social inertia to act [4].

**Figure 1.**
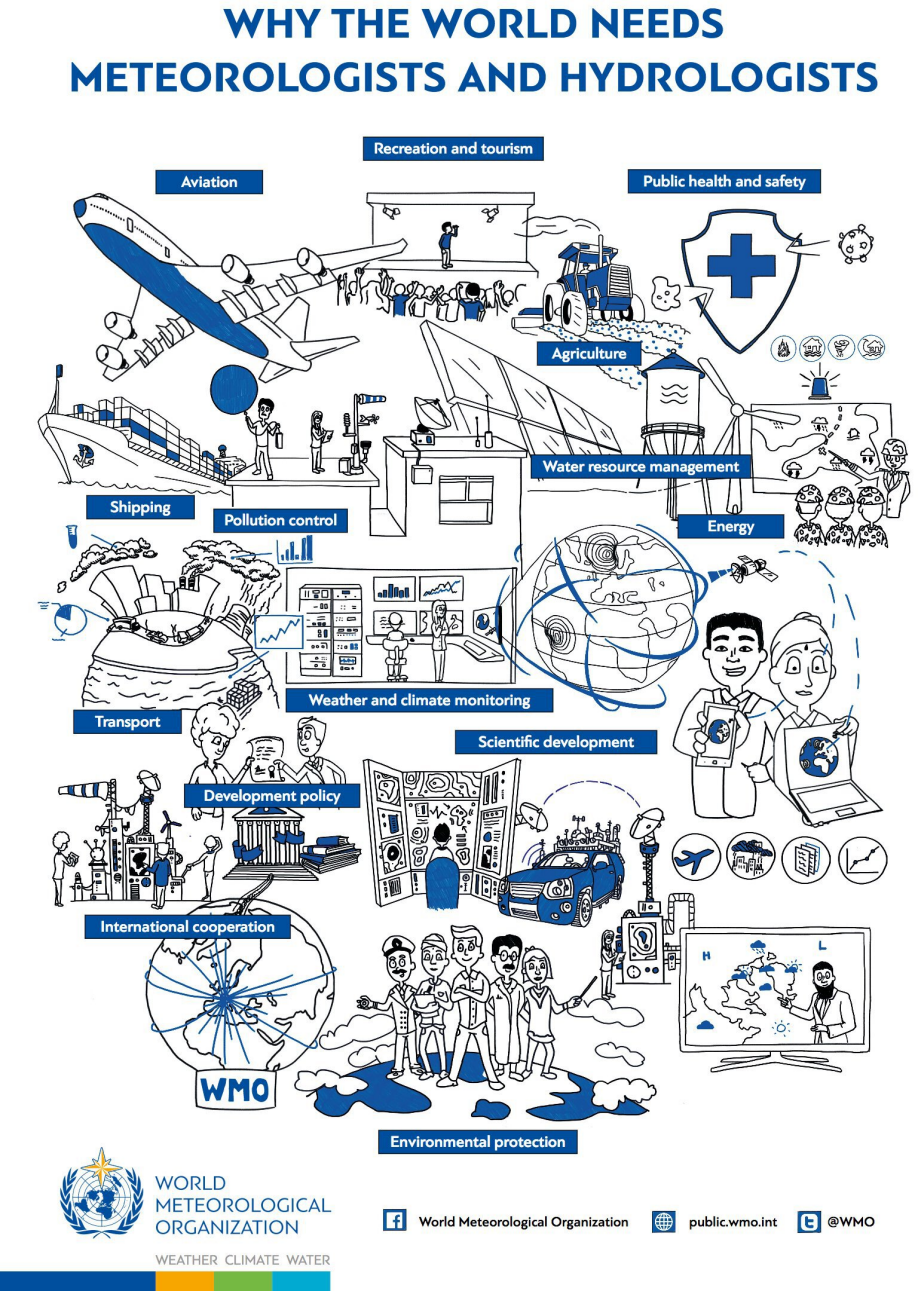
Why the world needs meteorologists and hydrologists. World Meteorological Organization [6].

Meeting the global political commitment to the UN Sustainable Development Goals [5] will require hydrological expertise in reducing the impact from water-related disasters (SDG11: sustainable cities and communities), improving water security (SDG1: no poverty; SDG2: zero hunger; SDG3: good health and wellbeing; SDG6: clear water and sanitation) and harnessing the power of water (SDG7: affordable and clean energy) [1]. Given water’s critical importance to society, hydrologists should be at the forefront of climate adaptation (figure 1), however, a recent survey of hydrologists in the UK [7] found that only 18% were satisfied with the value that society places on hydrology. A figure reflected in the low number of graduates embarking on hydrological careers [8]. There is an urgent need to improve the link between what we, as hydrologists, know is important and valuable work, and society’s perception of this. Joint initiatives such as the community identification of ‘Twenty-three unsolved problems in hydrology’ [9], the UK Flood Hydrology Roadmap which sets out a 25-year vision to ensure that society has improved hydrological information and understanding to manage flood hazard in a changing world [10], and the International Association of Hydrological Sciences (IAHS) identification of shared themes for hydrological research and practice over 10-year periods [11–13], attempt to address this need by speaking as one voice to raise awareness of the opportunities offered by hydrological research. There is an ongoing re-evaluation of the shape of hydrological science [14] and a collective focus on what hydrology can do to support solutions to global hydrological challenges in the current (2023–2032) IASH hydrological HELPING decade (Hydrology Engagement Local People IN one Global world, Arheimer *et al*. [13]).

Building on my own experiences in the field of forecasting and warning, this opinion paper explores the challenges and opportunities for closer links between hydrology and society. Despite the additional complexity of political, economic and social drivers in long-term risk management [4], there is much that can be learnt from the event forecasting timescale for the integration of hydrological knowledge into decision making at longer lead times. In event forecasting the decisions are immediate, there are opportunities for instant verification of response, and there are well-established communication channels between hydrologists and stakeholders. I believe the key is to focus on people—why hydrology matters to people and why people matter to (and in) hydrology. Throughout the paper people are regarded as individuals while ‘humans’ is used to refer to the characteristics of a group of people. People are considered in respect to four themes: people and hydrological science, people and hydrology education, people and hydrological decision making and people within the discipline of hydrology. These themes should be considered as interconnected, progress in any one of them alone is unlikely to result in greater societal uptake of hydrological knowledge.

## 2. People and hydrological science

The British Hydrological Society [15] defines hydrology as ‘the study of water in the environment’ going on to explain that ‘hydrology has evolved as a science to try and understand the complex water systems of the Earth, to study and predict how water will behave under different circumstances as it moves through the land phase of the water cycle.’ Hydrology as a distinct scientific discipline is relatively new. It emerged slowly in the early twentieth century from engineering roots with an initial focus on water supply, soil science and agriculture [16,17]. The establishment of groups such as the Hydrological Research Unit in the UK (later the Institute of Hydrology and now UKCEH) in the 1960s instigated an interdisciplinary focus, bringing together people with backgrounds in physics, mathematics, engineering, geography and geology [16] and considering hydrology within the more dynamic framing of earth system science [18]. In the early 2000s, new subdisciplines of hydrology started to develop that sought to explicitly integrate humans and water. Socio-hydrology considers the bidirectional feedbacks between humans and the water environment, hydrosociology strives to understand how human actions alter water systems and lead to human consequences, hydroeconomics aims to optimize the economic objectives of a water system and Integrated Water Resources Management has the overarching aim of managing water systems to reach desired outcomes for both society and the environment [19,20]. Similarly, the increasingly essential consideration of non-stationarity in hydrology [21,22] is a reflection on the influence of humans on hydrological systems through anthropogenic climate change and catchment management.

The Anthropocene discourse [23,24] recognized that human activity cannot be considered separately to natural processes. Humans are an intrinsic part of the hydrologic system, holding a central role as agents of change, beneficiaries of ecosystem services [14,25] and receptors of impacts from extreme events. However, humans are complex and the interface of hydrology with society remains on the list of unsolved problems in hydrology [9]. There have been multiple calls for interdisciplinary approaches to address this challenge and in particular for hydrology to foster closer integration with social scientists (e.g. [9,13,14,26,27]), I echo them but will not dwell on repeating them here. Progress in this direction is promising [28] but working across disciplines is hard and context specific, results are unlikely to be immediate and integrated modelling alone will not result in societal change. Instead, I focus on the vital role of people in research, education and practice as the link between hydrological science and hydrological decision making, and crucial to effective climate adaptation to water risks. In flood forecasting and warning, there has been a recent appreciation of the importance of co-designed systems, where people are not just passive receivers of information but an integral part of an effective forecasting chain [29] and crucial to developing a shared understanding of how science and data, alongside external drivers, shape decisions and action.

## 3. People and hydrology education

**Figure 2.**
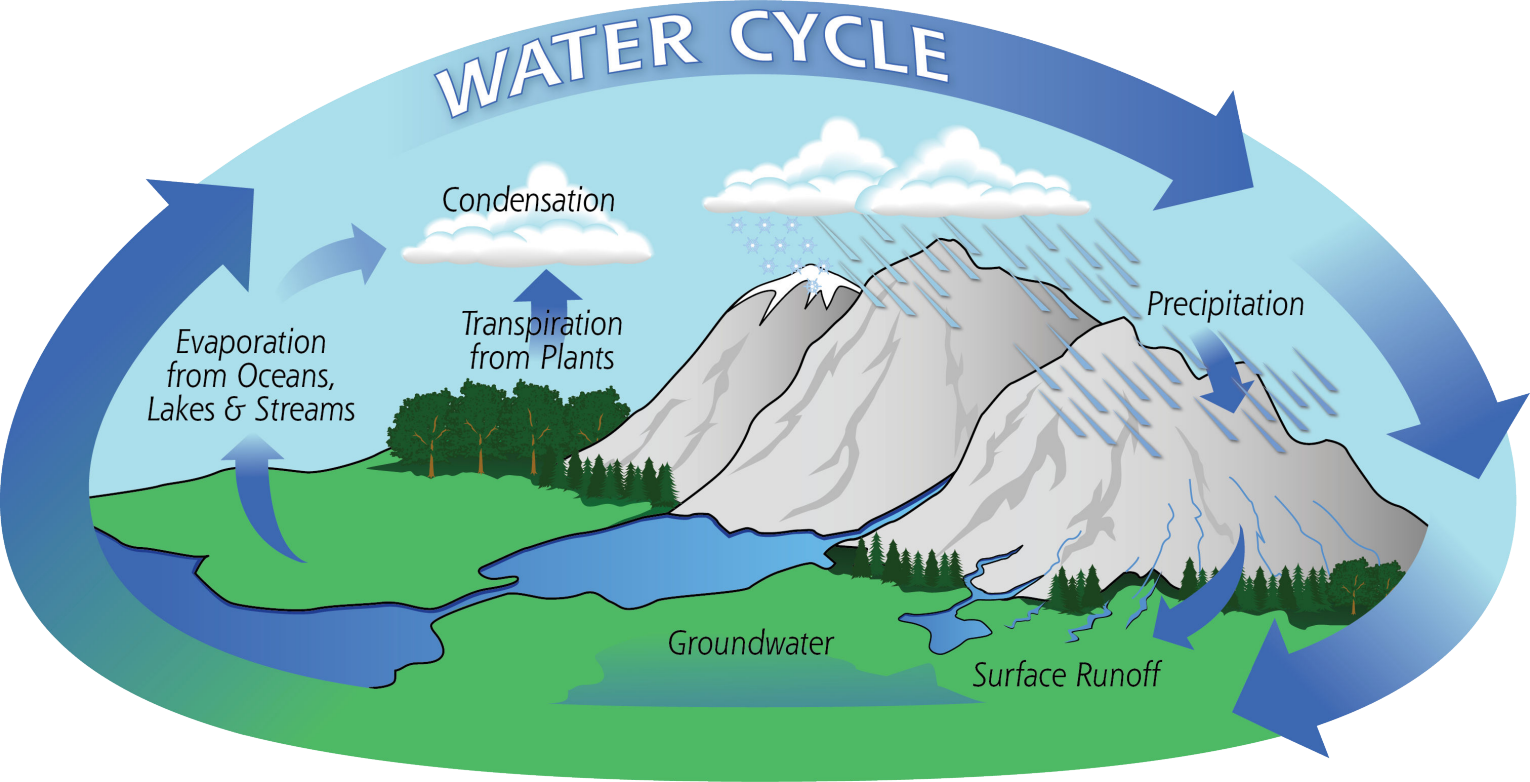
Diagram of the Water Cycle from NASA [31] that was returned as the top result from a Google Image search for 'The Water Cycle' in September 2024 illustrating the lack of consideration of humans.

**Figure 3.**
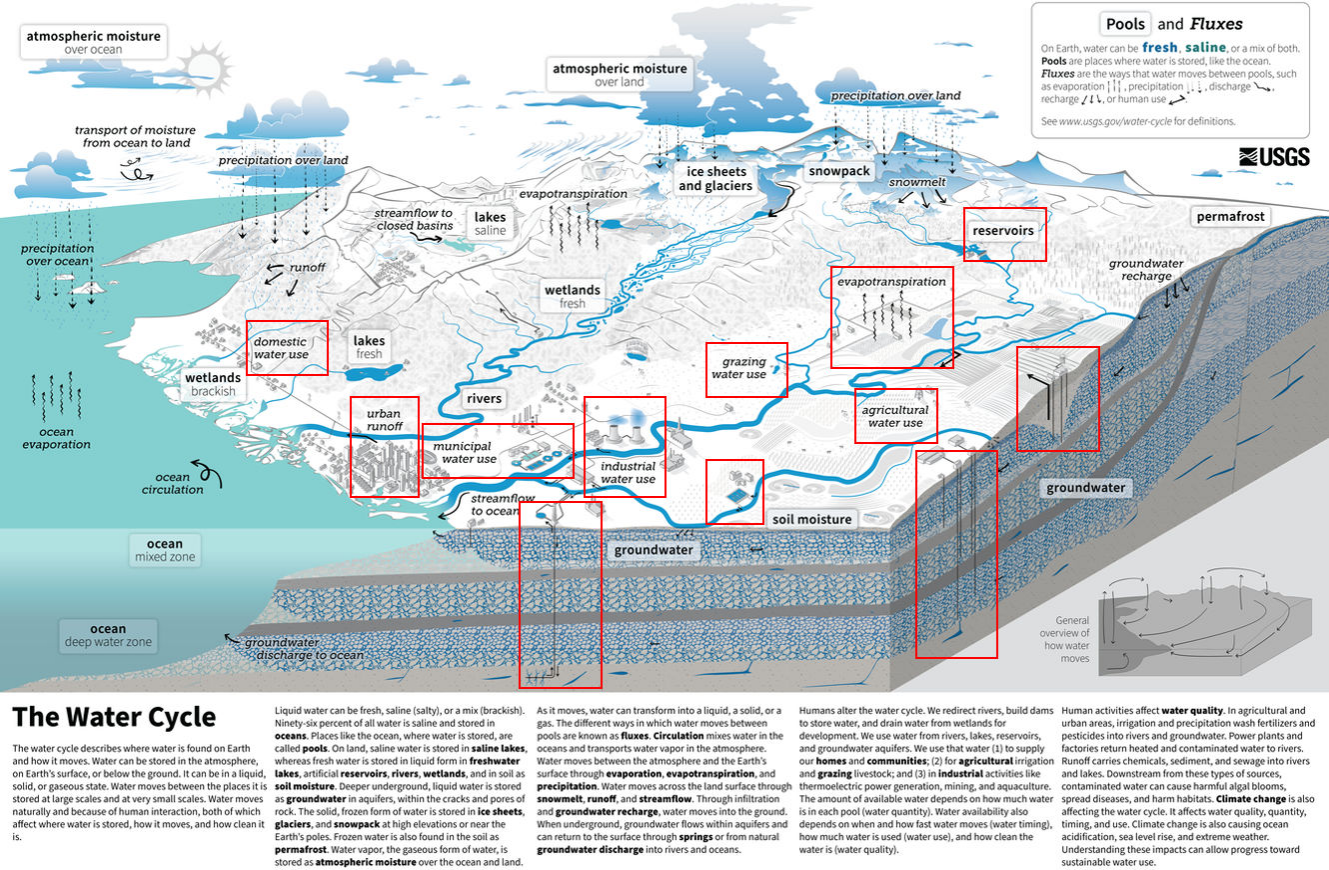
The new USGS Water Cycle diagram (with the explicit impacts of humans highlighted by the author in red boxes) [33].

Most people are introduced to hydrology through the water cycle. Abbot *et al*. [30] found that 85% of water cycle diagrams (for example, figure 2) showed no interactions between humans and hydrology. The reasons suggested for this included practicality (the need for simplification in diagrams), an aesthetic preference for natural landscapes, a remnant of the desire to establish hydrology as a natural science, or a lack of updating of traditional diagrams in the light of anthropogenic change. Regardless of the reason, if we are not evidencing the link between humans and hydrology in education and teaching at all levels then it is unsurprising that hydrology is not more centrally situated within decision making. As King *et al*. [32] argue, the next generation of problem solvers must be equipped with an understanding of the coupled hydrological, environmental, economic and social systems to tackle the unresolved hydrological challenges of the twenty-first century. In response to the findings of Abbott *et al*. [30], the US Geological Survey produced a new water cycle diagram (figure 3) and supporting educational materials [33] explicitly illustrating the impacts of human water and land use on water fluxes.

It is well documented [34–36] that access to more knowledge alone (the information deficit model) is an over-simplistic framework for understanding the link between evidence and decision making as it fails to account for the complex social, political and economic drivers of action. Here, my argument is that hydrological education that facilitate access to the building blocks to support people to understand how hydrology interacts with these wider drivers is an essential starting point to open up conversations on effective adaptation to hydrological risks. Hydrological education is also benefitting from an increasing focus on interdisciplinary approaches to teaching [37] and creative techniques [38,39] that bring to life the impact of hydrology on people.

## 4. People and hydrological decision making

In my own field of flood forecasting and warning, it is clear that despite ongoing scientific advances in the field, hydrological information is not leading to the action we, as expert hydrologists, can see is essential to protect lives and livelihoods before and during extreme events [40–44]. It seems unfathomable that in European countries with good forecasts and the capacity to act (§4a), over 200 people could die in the Storm Bernd floods of July 2021 across Germany, Belgium, the Netherlands and Luxembourg [44,45] or in Valencia, Spain in September 2024 [46]. Extreme events such as these are within the bounds of climate projections and can be forecasted by weather models. That does not mean that hydrometeorologists always get the details exactly right, there are issues with rapidly intensifying storms and translating from hazard to impact, but for scientists extreme events are not unexpected. Why then are people not heeding our advice? Why are governments and institutions not investing more in systems to support rapid response to flooding?

Part of the problem is access to information; following the flooding in Germany in 2021, Thieken *et al*. [44] found in a post-event survey that up to 35% of respondents in affected areas did not receive any form of warning. Another is perception of risk. A recent Environment Agency survey in England and Wales found that only 39% of people living in areas at risk of flooding believe their home could be at risk, and only 21% of people who received a flood warning felt concerned enough to want to take action [47]. Similarly, in Germany in 2021, of those who did receive a warning, 85% did not expect very severe flooding [44]. Having the best hydrological models in the world is no use if they are ignored. As Murphy [48, p. 286] observed ‘forecasts possess no intrinsic value. They acquire value through their ability to influence the decisions made by users of the forecast.’ Therefore, a new era of solutions-focused hydrology [13] must consider the human aspects of decision making.

Past experience of flooding strongly influences if, and how, people respond to risk information [47,49]. At policy level, major events often provide the impetus for structural change. Following the 2007 floods in the UK, the Pitt Review [50] was instrumental in establishing the joint Environment Agency and Met Office Flood Forecasting Centre and acted as a catalyst for improvements to probabilistic flood warning at lead times of up to five days [51]. More recently, surface water flooding in London in 2021 prompted a review of resilience to surface water flood risk [52,53] and provided momentum for collective action to improve surface water flood warning services [54]. There is a robust body of data and evidence that shows that the climate has changed and will continue to change, putting more people at risk from hydrological impacts [55]. In a rapidly changing climate, waiting for extreme events to initiate adaptation will be too late, decisions must be made on the basis of existing scientific evidence. This is not just a question of why others are not using our work, but also an opportunity for self-reflection and a reminder of why the work we do as hydrologists is important. In the era of twenty-first-century science, when we are busy seeking the next funding opportunity or chasing deadlines, it is easy to focus on our models and data and to skip the opportunities to reconnect with the catchments, communities and environments we work in. As Thompson [56, p. 7] asserts: ‘though Model Land is easy to enter, it is not so easy to leave’. Unless we, as hydrologists, can articulate the importance of our work, how will we encourage others to position hydrology more centrally within their decision making?

Drawing largely on the flood warning literature, here I outline four interlinked reasons why I believe hydrological expertise is not used more effectively in decision making at both individual and policy levels; capacity, complacency, complexity and communication.

### (a) Capacity

In many places, the capacity to think beyond the requirement for access to clean water supplies is limited by more immediate risks to life and health. Capacity to respond to long-term risk, or imminent threat, is limited by access to resources and political priorities. Capacity in this context is beyond the realm of hydrologists to solve alone (and beyond the scope of this paper). In these situations, hydrologists have a responsibility to act ethically to ensure that hydrological information is provided in user-friendly formats while acknowledging other priorities for decision making [57].

The climate adaptation literature [4] asserts that alongside risk assessments, successful adaptation must be supported by good governance and adequate financing. In the longer term, hydrologists can support capacity building by clearly communicating the urgency to act on hydrological risks to encourage improved governance and financing, enabling individuals, communities and organizations to be better able to respond to hydrological information.

### (b) Complacency

In their review of effective flood warning systems Kuller *et al*. [42, p. 2] observed that ‘people underestimate the risk posed by floods’ resulting in inadequate response to warnings. Flood risk communication encompasses two components: the identification of areas at risk of flooding and the communication of imminent threat [58], both are impacted by complacency. One reason for this is disconnection from the risk. Flood maps present an abstract representation of risk that is inaccessible to many people [59], existing flood defence systems may appear to be preforming well and flood risk is considered as something that only affects others, or for some risks like surface water flooding in urban areas there is no visible hazard (a river) to raise long-term awareness. Another challenge is dealing with ‘Black Swans’ [60], events that have not been experienced before and are therefore beyond the imagination of individuals or society [3,40,43]. In these situations, risk may be misrepresented, to borrow an example from outside hydrology recent heatwaves in both Canada [61] and the UK [62] were portrayed in the media as positive ‘beach days’ rather than a risk to life, or underplayed because people cannot imagine the magnitude of the event or how badly they will be affected [40].

Within forecasting and warning, a move towards impact-based warnings is advocated as a response to low-risk perception [42]. Impact-based warnings seek to encourage appropriate and timely actions by focusing on people-centred warnings of what the weather will do and how it will affect people, rather than what the weather will be [63]. Evidence of how successful impact-based warnings actually are in supporting proactive action remains mixed [63,64] and the underlying understanding of impacts at local and individual scales is limited by a lack of data and complex social contexts. Nonetheless there are valuable lessons on focusing on tangible, personally relevant impacts that could be translated more broadly to support the development of a more people-centred approach to communicating hydrological risks.

### (c) Complexity

Hydrology is complex with embedded uncertainties. Processes occur over multiple spatial and temporal scales, many outside the scales of observation networks. The system is highly interconnected requiring consideration of surface water, ground water, ecosystems, atmospheric and anthropogenic interactions. Non-stationarity means that the past is no longer a good predictor of the future [21]. Complexity makes hydrological decision making difficult. Here again there are parallels to flood forecast and warning. The use of ensembles in flood forecasting [65] requires the presentation of potentially complex probabilistic information, resulting in a growing appreciation of the challenges of incorporating this into decision making [66,67]. There is often a perception that people cannot understand probabilistic information, yet Ripberger *et al*. [68] found limited evidence for this in the literature, concluding that access to clearly presented probabilistic information improves the quality of decision making.

The first step is to consider what level of complexity is needed to support the users’ decisions [69,70]. Decision making is also complex, decisions are not made on any one source of information in isolation, and multiple users with different levels of hydrological understanding often make decisions based on the same product or information [71]. The trustworthiness of forecasts is higher when warnings are easier to understand [72], therefore additional complexity is frequently counterintuitive. In my own experience [71,73–75], being transparent about the strengths and limitations of the information provided, particularly when supported by an understanding of the local context, is important to support the effective communication of complex material.

### (d) Communication

Communicating the complexity and uncertainty in hydrology data, models, predictions and forecasts remains an unsolved problem in hydrology [9]. Much has been written about best practice for communicating uncertainty covering visualization, terminology and the level of detail that can be understood (e.g. [76–80]). Situated at the interface of Earth System Science and society, hydrologists should be well positioned to communicate effectively across disciplines, and with policymakers and the public. Respondents of the recent survey of hydrologists in the UK [7] identified ‘communicating with different audiences’ as the hydrological skill they were most confident in, illustrating that this is an oft-used skill of the twenty-first-century hydrologist, yet the lack of action on adaptation to hydrological risk indicates improvement in effective communication is still needed. Anderson *et al*. [81, p. 88] sum up the extent of the challenge well in their reflection on flood warnings: ‘in producing a warning the warner is as much as artist as a scientist, crafting a persuasive story out of a selection of uncertain facts, using their experience of context and precedent and fitting the results into a variety of formats to be delivered through different media, all while under considerable time pressure’.

In the field of flood warning, trust in communications is seen as crucial to determining response. Warning messages much be based on skilful forecasts and models appropriate for the users’ needs [70], credible [82] and perceived as trustworthy [83], as must the organizations that are delivering them [72]. The credibility of hydrology within society remains lower than hydrologists would like [7] creating barriers to effective action. In seeking to communicate effectively in a way that promotes action, hydrologists are also faced with complex data (§4c), competing messages from other media sources [84], competing priorities for resources (§4a) and events and concepts that are beyond the lived experience or imagination of decision makers (§4b). Creative approaches to communication are required that situate the message in a way that is relevant to individuals [85] and focus on the impacts of changes in the water cycle on people (§3).

## 5. People within the discipline of hydrology

The establishment of university courses in hydrology played an important role in establishing hydrology as an independent scientific discipline [17], but as the UK hydrology skills and satisfaction survey [7] shows, the use of hydrology skills and knowledge extends beyond those who would formally consider themselves as hydrologists. Hydrology in the twenty-first century needs hydrologists with the skills to work flexibly and innovatively with people from multiple disciplines and across the science-practice divide.

### (a) Interdisciplinary and user-focused hydrology

Responding to global water crises require people-focused, inter- and trans-disciplinary solutions [13,14]. To achieve this, improved two-way flow between hydrology and practice, and across local to global scales is needed [10,13,86], supported by accessible, reusable and reproducible research [87]. The skills now needed to do hydrology effectively are more numerous that any one person could, or should, be expected to obtain [7]. Therefore, hydrologists need to be effective communicators who seek to collaborate, and draw expertise from across disciplines (including across sub-disciplines of hydrology). In particular, people-focused hydrology calls for greater involvement of social and behavioural scientists to understand more about how people respond to hydrological information [54], and for hydrologists who actively strive to incorporate relevant findings from these fields into their own hydrological practice. It is not a new call [26,27], but the improved integration of social scientists remains an area that offers vast potential to ensure the value of hydrology to society is realized.

### (b) Rapidly evolving hydrological skills

Hydrology is a constantly evolving discipline. As such the skills of hydrologists must constantly evolve, allowing hydrologists flexibility to embrace new methods and opportunities. We are on the eve of another major change in hydrology, the opportunities presented by machine learning and big data [88] are allowing us to address the challenges of uncertainty and hydrology in data-sparse regions in new ways and across larger scales. Hydrologists will remain central to these endeavours demonstrating where, when and how hydrological knowledge adds value to machine learning models [89] by collaborating effectively with machine learning experts and developing machine learning skills within the discipline. Within hydrological forecasting the role of hydrologists is changing too. As forecasting models become more complicated, the opportunities to add value to the system are moving further along the forecasting chain [89–91], requiring new skills to enable hydrologists to effectively use their expertise in interpretation and communication of output with respect to knowledge of the local context.

### (c) Recognizing, valuing and developing the breadth of hydrological skills

It is not the case that *all* hydrologists need to develop skills in machine learning, communication, social science or any other aspect identified here. The breadth of skills within hydrology is a strength in addressing societal challenges. What is needed though is a clearer framework to develop, recognize and value the diversity within the discipline. The skills and time required for interdisciplinary working and communicating the value of hydrology to wider audiences are not easily rewarded in existing career structures and are not routinely embedded in hydrology training [92]. None the less progress is being made. Interdisciplinary doctoral training programmes [37] are proliferating, enabling students to study hydrology within the context of integrated Earth System Science, often with explicit goals of embedding students in innovative programmes that embrace new technologies and techniques to respond to real world challenges. For example Oxford University has two new programmes, the Interdisciplinary Life and Environmental Science Landscape Award (ILESLA) and Intelligent Earth (focused on AI for the Environment). National and international hydrological initiatives such as the IAHS HELPING decade and the UK Flood Hydrology Roadmap [10,13] provide a framework that support and showcase interdisciplinary approaches while raising the profile of hydrologists and building trust in hydrology. The voluntary engagement of hydrologists in initiatives such as these demonstrates the commitment from within the discipline to people-focused hydrology. To be successful, though, external funding and recognition are required to support continual training and development opportunities for new and established hydrologists.

## 6. Conclusion

As the discipline of hydrology has evolved, it has increasingly recognized the role of humans on the hydrological system, pushing hydrological science in new directions to study feedback between humans and the water environment. Yet, this article sought to explore a broader view of why people matter in hydrology, drawing from experience in flood forecasting and warning to explore the crucial link people play in the translation of hydrological science into practice. The collective ‘surprise’ [3,40,93] at the impacts of recent flood events across society shows that there is much more to be done to ensure hydrological risks are understood by people, and that governments and communities look to hydrological expertise to support adaptation to current and future water risks.

**Figure 4.**
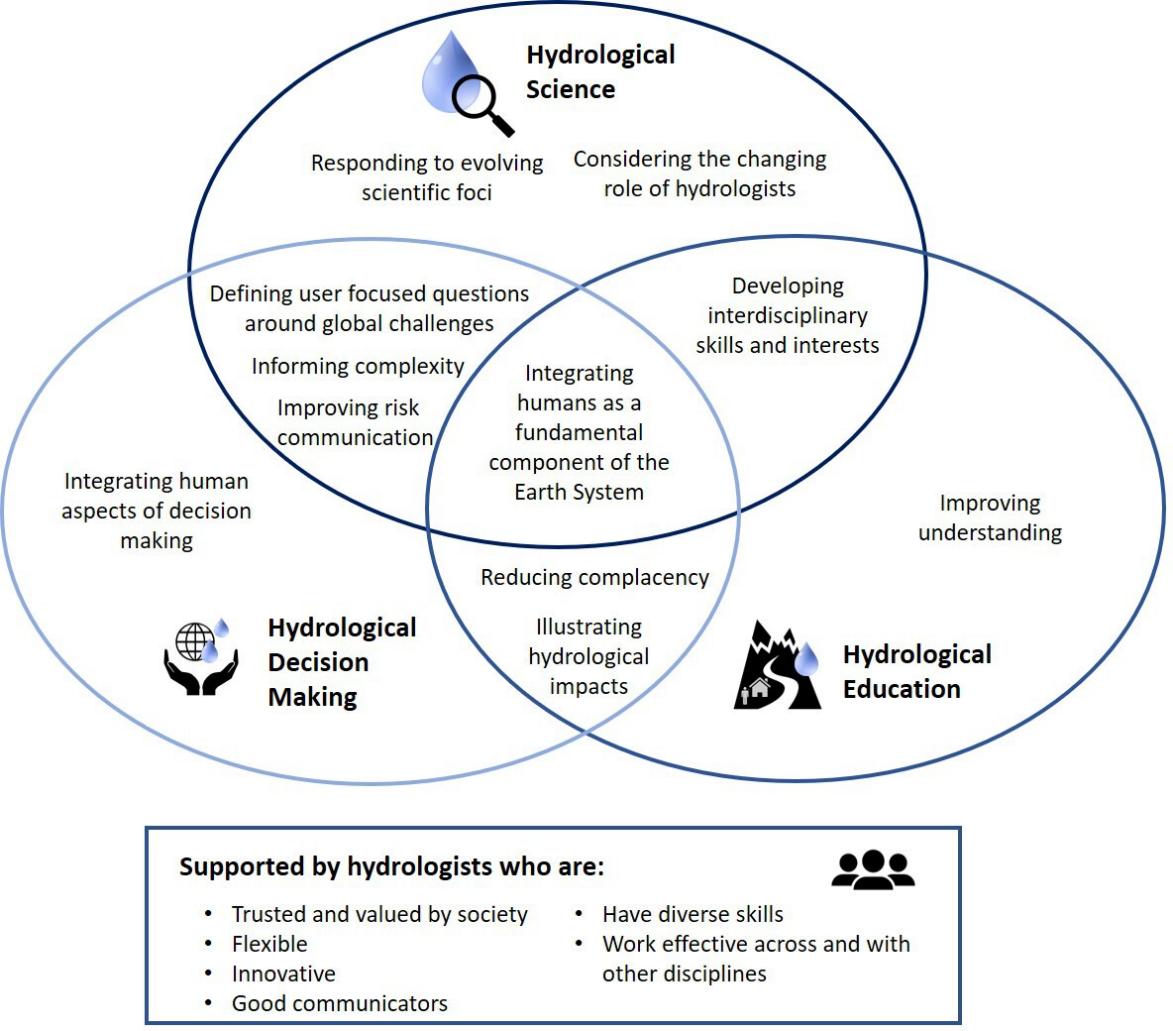
Intersecting areas where people matter in twenty-first-century hydrology.

The consideration of people in twenty-first-century hydrology (figure 4) must span hydrological education where more work is needed to ensure hydrology is taught in a way that highlights the relevance and impacts of hydrology to individuals, hydrological science where a focus on global hydrological challenges should inform user-focused interdisciplinary research, and hydrological decision making where hydrologists need to work closely with social scientists to understand why there is a disconnect between advances in hydrological science and improving resilience to water-related risks.

Hydrologists themselves are a principal asset of hydrology. As hydrology becomes more people-focused, the skill set required of hydrologists is changing. Alongside traditional hydrological skills, hydrologists for the twenty-first century must include good collaborators, people able to work flexibly across and between disciplines, people willing to incorporate knowledge from multiple fields into hydrological practice, and good communicators who can articulate the relevance of their work to others. Hydrology must seek to value the diversity of skills within the discipline as the key to its success.

I firmly believe that the strength of our hydrological data, models and expertise are good enough to support effective climate adaptation. As a discipline, hydrology must put more focus on how knowledge is communicated, the best forecasting models in the world are no use if people do not take action based on the forecast.

## Data Availability

This article has no additional data.

## References

[1] Caretta MA. 2022 Water. In Climate change 2022: impacts, adaptation and vulnerability. Contribution of working group II to the sixth assessment report of the intergovernmental panel on climate change (eds KM Poloczanska, A Alegría, M Craig, S Langsdorf, S Löschke, V Möller, A Okem, B Rama), pp. 551–712. Cambridge, UK and New York, NY: Cambridge University Press. (10.1017/9781009325844.006)

[2] World Weather Attribution. 2024 Extreme weather attribution study tracker. See https://www.worldweatherattribution.org/study-tracker/ (accessed 1 December 2024).

[3] Heinrich D, Stephens E, Perez E. 2024 More than magnitude: towards a multidimensional understanding of unprecedented weather to better support disaster management. Water Secur **23**, 100181. (10.1016/j.wasec.2024.100181)

[4] Aerts JCJH, Bates PD, Botzen WJW. 2024 Exploring the limits and gaps of flood adaptation. Nat. Water **2**, 719–728. (10.1038/s44221-024-00274-x)

[5] United Nations. 2015 Transforming our world: the 2030 agenda for sustainable development. See https://sdgs.un.org/sites/default/files/publications/21252030%20Agenda%20for%20Sustainable%20Development%20web.pdf (accessed 1 April 2025).

[6] World Meteorological Organisation. 2017 Why the world needs meteorologists. See https://library.wmo.int/idurl/4/54014 (accessed 1 December 2024).

[7] Environment Agency. 2024 UK hydrology skills and satisfaction survey, 2023: the results. See https://engageenvironmentagency.uk.engagementhq.com /w4-skills-survey (accessed 10 October 2024).

[8] Faulkner D. 2025 President’s perspective. Circulation, British Hydrological Society. See https://www.hydrology.org.uk/dms-files.php?id=1591&action=doc (accessed 1 April 2025).

[9] Blöschl G *et al*. 2019 Twenty-three unsolved problems in hydrology (UPH) – a community perspective. Hydrol. Sci. J. **64**, 1141–1158. (10.1080/02626667.2019.1620507)

[10] Lamb R *et al*. 2022 The future of flood hydrology in the UK. Hydrol. Res. **53**, 1286–1303. (10.2166/nh.2022.053)

[11] Sivapalan M. 2003 IAHS Decade on predictions in ungauged basins (PUB), 2003–2012: shaping an exciting future for the hydrological sciences. Hydrol. Sci. J. **48**, 857–880. (10.1623/hysj.48.6.857.51421)

[12] Montanari A *et al*. 2013 Panta Rhei—everything flows: change in hydrology and society—the IAHS scientific decade 2013–2022. Hydrol. Sci. J. **58**, 1256–1275. (10.1080/02626667.2013.809088)

[13] Arheimer B, Cudennec C, Castellarin A, Grimaldi S, Heal KV, Lupton C. 2024 The IAHS science for solutions decade, with hydrology engaging local people in one global world (HELPING). Hydrol. Sci. J. **69**, 1417–1435. (10.1080/02626667.2024.2355202)

[14] Wagener T *et al*. 2010 The future of hydrology: an evolving science for a changing world. Water Resour. Res. **46**, R008906. (10.1029/2009wr008906)

[15] British Hydrological Society. The science of hydrology. See https://www.hydrology.org.uk/science_of_hydrology.php (accessed 10 October 2024).

[16] McCulloch C. 2022 Dramatic growth of hydrological science in the UK in the 1960s and early 1970s. Hydrol. Res. **53**, 754–765. (10.2166/nh.2022.005)

[17] Rodda JC. 2006 On the British contribution to international hydrology—an historical perspective. Hydrol. Sci. J. **51**, 1177–1193. (10.1623/hysj.51.6.1177)

[18] Sivapalan M. 2018 From engineering hydrology to Earth system science: milestones in the transformation of hydrologic science. Hydrol. Earth Syst. Sci. **22**, 1665–1693. (10.5194/hess-22-1665-2018)

[19] Pande S, Sivapalan M. 2017 Progress in socio‐hydrology: a meta‐analysis of challenges and opportunities. WIREs Water **4**, e1193. (10.1002/wat2.1193)

[20] Sivapalan M, Savenije HHG, Blöschl G. 2012 Socio‐hydrology: a new science of people and water. Hydrol. Process. **26**, 1270–1276. (10.1002/hyp.8426)

[21] Milly PCD, Betancourt J, Falkenmark M, Hirsch RM, Kundzewicz ZW, Lettenmaier DP, Stouffer RJ. 2008 Stationarity is dead: whither water management? Science **319**, 573–574. (10.1126/science.1151915) 18239110

[22] Slater LJ *et al*. 2021 Nonstationary weather and water extremes: a review of methods for their detection, attribution, and management. Hydrol. Earth Syst. Sci. **25**, 3897–3935. (10.5194/hess-25-3897-2021)

[23] Lewis SL, Maslin MA. 2015 Defining the Anthropocene. Nature **519**, 171–180. (10.1038/nature14258) 25762280

[24] Steffen W, Grinevald J, Crutzen P, McNeill J. 2011 The Anthropocene: conceptual and historical perspectives. Phil. Trans. R. Soc. A **369**, 842–867. (10.1098/rsta.2010.0327) 21282150

[25] Vogel RM, Lall U, Cai X, Rajagopalan B, Weiskel PK, Hooper RP, Matalas NC. 2015 Hydrology: the interdisciplinary science of water. Water Resour. Res. **51**, 4409–4430. (10.1002/2015wr017049)

[26] Haggett C. 1998 An integrated approach to flood forecasting and warning in England and Wales. Water Environ. J. **12**, 425–432. (10.1111/j.1747-6593.1998.tb00211.x)

[27] Ratna Reddy V, Syme GJ. 2014 Social sciences and hydrology: an introduction. J. Hydrol. **518**, 1–4. (10.1016/j.jhydrol.2014.06.022)

[28] Rahman M, Frame JM, Lin J, Nearing GS. 2022 Hydrology research articles are becoming more topically diverse. J. Hydrol. **614**, 128551. (10.1016/j.jhydrol.2022.128551)

[29] Golding B (ed). 2022 Towards the ‘perfect’ weather warning. Cham, Switzerland: Springer.

[30] Abbott BW, Bishop K, Zarnetske JP. 2019 Human domination of the global water cycle absent from depictions and perceptions. Nat. Geosci. **12**, 533–540. (10.1038/s41561-019-0374-y)

[31] NASA. Precipitation education: the water cycle. See https://gpm.nasa.gov/education/water-cycle (accessed 1 December 2024).

[32] King EG, O’Donnell FC, Caylor KK. 2012 Reframing hydrology education to solve coupled human and environmental problems. Hydrol. Earth Syst. Sci. **16**, 4023–4031. (10.5194/hess-16-4023-2012)

[33] USGS. 2023 A new take on the water cycle. See https://waterdata.usgs.gov/blog/water-cycle-release/ (accessed 1 December 2024).

[34] Ecker UKH, Lewandowsky S, Cook J, Schmid P, Fazio LK, Brashier N, Kendeou P, Vraga EK, Amazeen MA. 2022 The psychological drivers of misinformation belief and its resistance to correction. Nat. Rev. Psychol. **1**, 13–29. (10.1038/s44159-021-00006-y)

[35] Junier SJ. 2017 Experts and expertise in the implementation of the Water Framework Directive in The Netherlands. Doctoral Thesis, TU Delft, The Netherlands. 10.4233/uuid:eea8a911-f786-4158-a67e-b99663275bf8.

[36] Suldovsky B. 2017 The Information Deficit Model and Climate Change Communication. Oxford Research Encyclopedia of Climate Science. See https://oxfordre.com/climatescience/view/10.1093/acrefore/9780190228620.001.0001/acrefore-9780190228620-e-301 (accessed 1 April 2025).

[37] Wagener T *et al*. 2021 Hydroinformatics education – the Water Informatics in Science and Engineering (WISE) Centre for Doctoral Training. Hydrol. Earth Syst. Sci. **25**, 2721–2738. (10.5194/hess-25-2721-2021)

[38] Parsons K, Lloyd Williams A, Skinner C. 2025 Using 360° immersive storytelling to engage communities with flood risk. Geogr. Res. **63**, 91–102. (10.1111/1745-5871.12682)

[39] Skinner C. 2020 Flash flood!: a SeriousGeoGames activity combining science festivals, video games, and virtual reality with research data for communicating flood risk and geomorphology. Geosci. Commun. **3**, 1–17. (10.5194/gc-3-1-2020)

[40] Cloke H. 2022 Science needs to address its imagination problem – lives depend on it, NewScientist. See https://www.newscientist.com/article/mg25333753-500-science-needs-to-address-its-imagination.

[41] O’Donnell EC, Thorne CR. 2020 Drivers of future urban flood risk. Phil. Trans. R. Soc. A **378**, 20190216. (10.1098/rsta.2019.0216) 32063161 PMC7061970

[42] Kuller M, Schoenholzer K, Lienert J. 2021 Creating effective flood warnings: a framework from a critical review. J. Hydrol. **602**, 126708. (10.1016/j.jhydrol.2021.126708)

[43] Ommer J, Neumann J, Kalas M, Blackburn S, Cloke HL. 2024 Surprise floods: the role of our imagination in preparing for disasters. Nat. Hazards Earth Syst. Sci. **24**, 2633–2646. (10.5194/nhess-24-2633-2024)

[44] Thieken AH, Bubeck P, Heidenreich A, von Keyserlingk J, Dillenardt L, Otto A. 2023 Performance of the flood warning system in Germany in July 2021 – insights from affected residents. Nat. Hazards Earth Syst. Sci. **23**, 973–990. (10.5194/nhess-23-973-2023)

[45] Szönyi M, Roezer V, Deubelli T, Ulrich J, MacClune K, Laurien F, Norton R. 2022 PERC Flood event review ‘Bernd'. Zurich Insurance Company. See https://zcralliance.org/resources/item/perc-flood-event-review-bernd/ (accessed 1 December 2024).

[46] The Guardian. 2024 Almost half of Valencia’s flood victims were aged over 70, figures show. See https://www.theguardian.com/world/2024/nov/14/almost-half-of-spains-flood-victims-were-over-70-figures-show#:~:text=A%20total%20of%20224%20people,the%20largest%20losses%20of%20life (accessed 1 December 2024).

[47] Environment Agency. 2023 Public flood survey 2023. Internal report.

[48] Murphy AH. 1993 What is a good forecast? An essay on the nature of goodness in weather forecasting. Weather Forecast. **8**, 281–293. (10.1175/1520-0434(1993)0082.0.co;2)

[49] Kox T, Thieken AH. 2017 To act or not to act? Factors influencing the general public’s decision about whether to take protective action against severe weather. Wea. Clim. Soc. **9**, 299–315. (10.1175/WCAS-D-15-0078.1)

[50] Pitt M. 2008 Learning lessons from the 2007 floods. An independent review by Sir Michael Pitt; Cabinet Office: London, UK. See http://archive.cabinetoffice.gov.uk/pittreview/thepittreview.html.

[51] Pilling C, Dodds V, Cranston M, Price D, Harrison T, How A. 2016 Chapter 9 - Flood forecasting — a national overview for Great Britain. In Flood forecasting (eds TE Adams, TC Pagano), pp. 201–247. London, UK: Academic Press. (10.1016/B978-0-12-801884-2.00009-8)

[52] Mayor of London. 2022 Surface water flooding in London: roundtable progress report, p. 44. London, UK: Greater London Authority, City Hall. See https://www.london.gov.uk/sites/default/files/flooding_progress_report_final_1.pdf.

[53] National Infrastructure Commission. 2022 Reducing the risk of surface water flooding, p. 72. See https://nic.org.uk/app/uploads/NIC-Reducing-the-Risk-of-Surface-Water-Flooding-Final-28-Nov-2022.pdf.

[54] Speight L, Birch CE, Self K, Brown S. 2025 Expert perspectives on the next generation of UK surface water flood warning services. Weather (10.1002/wea.7724)

[55] IPCC. 2021 Climate change 2021: the physical science basis. contribution of working group I to the sixth assessment report of the intergovernmental panel on climate change, (eds TK Maycock, T Waterfield, O Yelekçi, R Yu, B Zhou), p. 2391. Cambridge, UK and New York, NY: Cambridge University Press. (10.1017/9781009157896)

[56] Thompson E. 2022 Escape from model land. London, UK: Basic Books.

[57] Hawker L, Trigg MA, Kruczkiewicz A, Bernhofen M, Katsi L, Paterson R, Speight L, Den Hoek J, Balfour N. 2024 Data, guidelines and ethics for managing flood risk when people are already forcibly displaced. Environ. Res. Lett. **20**. (10.1088/1748-9326/ad9e06)

[58] Rollason E, Bracken LJ, Hardy RJ, Large ARG. 2018 Rethinking flood risk communication. Nat. Hazards **92**, 1665–1686. (10.1007/s11069-018-3273-4)

[59] Percival SE, Gaterell M, Hutchinson D. 2020 Effective flood risk visualisation. Nat. Hazards **104**, 375–396. (10.1007/s11069-020-04173-8)

[60] Taleb NN. 2007 The black swan: the impact of the highly improbable. London, UK: Allen Lane.

[61] Tetzlaff EJ, Goulet N, Yapici N, Gorman M, Richardson GRA, Enright PM, Kenny GP. 2024 Beach day or deadly heatwave? Content analysis of media images from the 2021 Heat Dome in Canada. Clim. Chang. **177**, 74. (10.1007/s10584-024-03713-6)

[62] Carbon Brief. 2022 Media reaction: UK’s record-smashing 40c heatwave and climate change. See https://www.carbonbrief.org/media-reaction-uks-record-smashing-40c-heatwave-and-climate-change/ (accessed 1 December 2024).

[63] Potter S, Harrison S, Kreft P. 2021 The benefits and challenges of implementing impact-based severe weather warning systems: perspectives of weather, flood, and emergency management personnel. Flood Emerg. Manag. Pers. Wea Clim. Soc. **13**, 303–314. (10.1175/WCAS-D-20-0110.1)

[64] Potter SH, Kreft PV, Milojev P, Noble C, Montz B, Dhellemmes A, Woods RJ, Gauden-Ing S. 2018 The influence of impact-based severe weather warnings on risk perceptions and intended protective actions. Int. J. Disaster Risk Reduct. **30**, 34–43. (10.1016/j.ijdrr.2018.03.031)

[65] Cloke HL, Pappenberger F. 2009 Ensemble flood forecasting: a review. J. Hydrol. **375**, 613–626. (10.1016/j.jhydrol.2009.06.005)

[66] Arnal L, Anspoks L, Manson S, Neumann J, Norton T, Stephens E, Wolfenden L, Cloke HL. 2020 ‘Are we talking just a bit of water out of bank? Or is it Armageddon?’ Front line perspectives on transitioning to probabilistic fluvial flood forecasts in England. Geosci. Commun. **3**, 203–232. (10.5194/gc-3-203-2020)

[67] Coughlan de Perez E *et al*. 2016 Action-based flood forecasting for triggering humanitarian action. Hydrol. Earth Syst. Sci. **20**, 3549–3560. (10.5194/hess-20-3549-2016)

[68] Ripberger J, Bell A, Fox A, Forney A, Livingston W, Gaddie C, Silva C, Jenkins-Smith H. 2022 Communicating probability information in weather forecasts: findings and recommendations from a living systematic review of the research literature. Wea Clim. Soc. **14**, 481–498. (10.1175/WCAS-D-21-0034.1)

[69] Scolobig A *et al*. 2022 Connecting warning with decision and action: a partnership of communicators and users. In Towards the ‘perfect’ weather warning (ed. B Golding), pp. 47–85. Cham, Switzerland: Springer. (10.1007/978-3-030-98989-7_3)

[70] Hossain S, Cloke HL, Ficchì A, Gupta H, Speight L, Hassan A, Stephens EM. 2025 A decision‐led evaluation approach for flood forecasting system developments: an application to the Global Flood Awareness System in Bangladesh. J. Flood Risk Manag **18**, e12959. (10.1111/jfr3.12959)

[71] Speight L *et al*. 2025 Recommendations to improve the interpretation of global flood forecasts to support international humanitarian operations for tropical cyclones. J. Flood Risk Manag. **18**, e12952. (10.1111/jfr3.12952)

[72] Taylor AL, Kause A, Summers B, Harrowsmith M. 2019 Preparing for Doris: exploring public responses to impact-based weather warnings in the United Kingdom. Weather. Clim. Soc. **11**, 713–729. (10.1175/WCAS-D-18-0132.1)

[73] Budimir M, Sneddon A, Nelder I, Brown S, Donovan A, Speight L. 2022 Development of forecast information for institutional decision-makers: landslides in India and cyclones in Mozambique. Geosci. Commun. **5**, 151–175. (10.5194/gc-5-151-2022)

[74] Emerton R *et al*. 2020 Emergency flood bulletins for cyclones Idai and Kenneth: a critical evaluation of the use of global flood forecasts for international humanitarian preparedness and response. Int. J. Disast. Risk Re **50**, 1–30. (10.1016/j.ijdrr.2020.101811)

[75] Speight L *et al*. 2018 Developing surface water flood forecasting capabilities in Scotland: an operational pilot for the 2014 Commonwealth Games in Glasgow. J. Flood Risk Manag. **11**, S884–S901. (10.1111/jfr3.12281)

[76] Beven K, Lamb R, Leedal D, Hunter N. 2015 Communicating uncertainty in flood inundation mapping: a case study. Int. J. River Basin Manag. **13**, 2014. (10.1080/15715124.2014.917318)

[77] McMillan HK, Westerberg IK, Krueger T. 2018 Hydrological data uncertainty and its implications. WIREs Water **5**, e1319. (10.1002/wat2.1319)

[78] Ramos MH, Mathevet T, Thielen J, Pappenberger F. 2010 Communicating uncertainty in hydro-meteorological forecasts: mission impossible? Met. Apps **17**, 223–235. (10.1002/met.202)

[79] Stephens EM, Spiegelhalter DJ, Mylne K, Harrison M. 2019 The met office weather game: investigating how different methods for presenting probabilistic weather forecasts influence decision-making. Geosci. Commun. **2**, 101–116. (10.5194/gc-2-101-2019)

[80] Taylor A, Summers B, Domingos S, Garrett N, Yeomans S. 2024 The effect of likelihood and impact information on public response to severe weather warnings. Risk Anal. **44**, 1237–1253. (10.1111/risa.14222) 37743536

[81] Anderson CL *et al*. 2022 Connecting forecast and warning: a partnership between communicators and scientists. In Towards the ‘perfect’ weather warning (ed. B Golding), pp. 87–113. Cham, Switzerland: Springer International Publishing. (10.1007/978-3-030-98989-7_4)

[82] Perreault MF, Houston JB, Wilkins L. 2014 Does scary matter?: testing the effectiveness of new National Weather Service tornado warning messages. Commun. Stud. **65**, 484–499. (10.1080/10510974.2014.956942)

[83] Losee JE, Joslyn S. 2018 The need to trust: how features of the forecasted weather influence forecast trust. Int. J. Disaster Risk Reduct. **30**, 95–104. (10.1016/j.ijdrr.2018.02.032)

[84] Parida D, Moses S, Rahaman KR. 2021 Analysing media framing of cyclone Amphan: implications for risk communication and disaster preparedness. Int. J. Disaster Risk Reduct. **59**, 102272. (10.1016/j.ijdrr.2021.102272)

[85] Hayhoe K. 2022 Saving us: a climate scientist’s case for hope and healing in a divided world. New York, NY: One Signal Publishers.

[86] van Hateren TC *et al*. 2023 Where should hydrology go? An early-career perspective on the next IAHS Scientific Decade: 2023–2032. Hydrol. Sci. J. **68**, 529–541. (10.1080/02626667.2023.2170754)

[87] Hall CA, Saia SM, Popp AL, Dogulu N, Schymanski SJ, Drost N, van Emmerik T, Hut R. 2022 A hydrologist’s guide to open science. Hydrol. Earth Syst. Sci. **26**, 647–664. (10.5194/hess-26-647-2022)

[88] Slater LJ. 2025 Challenges and opportunities of ML and explainable AI in large-sample hydrology. Phil Trans R. Soc. A **383**, 20240287. (10.1098/rsta.2024.0287) 40739919 PMC12334205

[89] Nearing GS, Kratzert F, Sampson AK, Pelissier CS, Klotz D, Frame JM, Prieto C, Gupta HV. 2021 What role does hydrological science play in the age of machine learning? Water Resour. Res. **57**, R028091. (10.1029/2020wr028091)

[90] Stuart NA *et al*. 2022 The evolving role of humans in weather prediction and communication. Bull. Am. Meteorol. Soc. **103**, E1720–E1746. (10.1175/BAMS-D-20-0326.1)

[91] Speight LJ, Cranston MD, White CJ, Kelly L. 2021 Operational and emerging capabilities for surface water flood forecasting. WIREs Water **8**, e1517. (10.1002/wat2.1517)

[92] Brown RR, Deletic A, Wong THF. 2015 Interdisciplinarity: how to catalyse collaboration. Nature **525**, 315–317. (10.1038/525315a) 26381970

[93] Kelder T *et al*. 2025 How to stop being surprised by unprecedented weather. Nat. Commun. **16**, 2382. (10.1038/s41467-025-57450-0) 40064852 PMC11894206

